# Enhanced Photocatalytic Activity for H_2_ Evolution under Irradiation of UV–Vis Light by Au-Modified Nitrogen-Doped TiO_2_

**DOI:** 10.1371/journal.pone.0103671

**Published:** 2014-08-04

**Authors:** Weirong Zhao, Zhuyu Ai, Jiusong Dai, Meng Zhang

**Affiliations:** Department of Environmental Engineering, Zhejiang University, Hangzhou, China; Argonne National Laboratory, United States of America

## Abstract

**Background Purpose:**

Photocatalytic water splitting for hydrogen evolution is a potential way to solve many energy and environmental issues. Developing visible-light-active photocatalysts to efficiently utilize sunlight and finding proper ways to improve photocatalytic activity for H_2_ evolution have always been hot topics for research. This study attempts to expand the use of sunlight and to enhance the photocatalytic activity of TiO_2_ by N doping and Au loading.

**Methods:**

Au/N-doped TiO_2_ photocatalysts were synthesized and successfully used for photocatalytic water splitting for H_2_ evolution under irradiation of UV and UV–vis light, respectively. The samples were characterized using X-ray diffraction (XRD), transmission electron microscopy (TEM), X-ray photoelectron spectroscopy (XPS), UV–vis diffuse reflectance spectroscopy (DRS), photoluminescence spectroscopy (PL), and photoelectrochemical characterizations.

**Results:**

DRS displayed an extension of light absorption into the visible region by doping of N and depositing with Au, respectively. PL analysis indicated electron-hole recombination due to N doping and an efficient inhibition of electron-hole recombination due to the loaded Au particles. Under the irradiation of UV light, the photocatalytic hydrogen production rate of the as-synthesized samples followed the order Au/TiO_2_ > Au/N-doped TiO_2_ > TiO_2_ > N-doped TiO_2_. While under irradiation of UV–vis light, the N-TiO_2_ and Au/N-TiO_2_ samples show higher H_2_ evolution than their corresponding nitrogen-free samples (TiO_2_ and Au/TiO_2_). This inconsistent result could be attributed to the doping of N and the surface plasmonic resonance (SPR) effect of Au particles extending the visible light absorption. The photoelectrochemical characterizations further indicated the enhancement of the visible light response of Au/N-doped TiO_2_.

**Conclusion:**

Comparative studies have shown that a combination of nitrogen doping and Au loading enhanced the visible light response of TiO_2_ and increased the utilization of solar energy, greatly boosting the photocatalytic activity for hydrogen production under UV–vis light.

## Introduction

Energy and environmental issues have become the focus of world attention. Developing clean and renewable energy is a crucial step in solving these issues. The conversion of solar energy to hydrogen via photocatalytic water splitting presents a promising alternative for a renewable, clean and economical process [1]–[3]. Exploiting visible-light-active photocatalysts for the efficient utilization of sunlight and finding proper ways to improve photocatalytic activity for H_2_ evolution are still the major challenges preventing photocatalytic water splitting, therefore, receive a lot of attention from researchers.

N-doped TiO_2_ has been widely studied because of its ability to extend the light absorption of TiO_2_ into the visible region [4], [5]. It was reported that the localized states introduced via nitrogen doping and simultaneously induced oxygen vacancies were responsible for the visible light photoactivity [6]. However, these impurity levels may enhance the recombination rate of photogenerated electrons and holes, thus decreasing the photocatalytic activity [7], [8]. As one of the most promising methods to improve photocatalytic efficiency, loading of noble metals, such as Au, Ag, or Pt, has also been extensively studied [9]–[12]. These noble metals can effectively promote the transfer of photoinduced electrons due to the formation of a Schottky barrier at the interface between the metal and the semiconductor, thereby reducing the recombination of photogenerated charge carriers, resulting in an enhancement of the photocatalytic activity. Meanwhile, Au-loaded samples have attracted broad attention [13] because it is a plasmonic metal with a surface plasmonic resonance (SPR) effect that supposedly has the ability to increase visible light photocatalytic efficiency, e.g., Au-loaded TiO_2_ [14] and Au-loaded ZnO [15].

Based on the specific features of nitrogen doping and Au loading, it is possible to compensate for the deficiencies in the N doping by Au loading, thereby expanding the utilization of solar energy and boosting the photocatalytic activity for hydrogen evolution. Sanz et al. [16] reported that Au adsorption on N-doped TiO_2_ (Au/N-TiO_2_) surfaces led to a higher stabilization of N species and a synergic effect between implanted N and deposited Au atoms. The Au/TiN*_x_*O_2-*y*_ system was able to catalyze hydrogen evolution via the water-gas shift reaction at elevated temperatures (575–625 K). Tian et al. [17] adopted a simple wet-chemical method to prepare Au/N-TiO_2_ photocatalysts, which exhibited much higher visible-light photocatalytic activity than N-doped or Au-loaded TiO_2_. Wu et al. [18] reported that Au/N-TiO_2_ exhibited a much higher photocatalytic activity in the degradation of methyl orange compared to N-TiO_2_ due to the appropriate sizes of Au particles and the synergic effect between the N dopant and the Au particles. Despite the current progress, the exploration of Au/N-TiO_2_ photocatalysts, especially for their application in photocatalytic water splitting for H_2_ generation, is still very necessary.

In the present work, the photocatalytic water splitting for H_2_ generation by TiO_2_, N-doped TiO_2_, Au-loaded TiO_2_ and Au-modified N-doped TiO_2_ was compared under irradiation of UV and UV–vis light. The photocatalytic mechanism of Au/N-TiO_2_ was further investigated using DRS, PL, and photoelectrochemical characterization.

## Experimental Section

### Chemicals and sample preparation

P25 (Degussa, Germany) was used in the preparation of TiO_2_ and N-doped TiO_2_. Chloroauric acid tetrahydrate (AuCl_3_•HCl•4H_2_O), sodium hydroxide (NaOH), nitric acid (HNO_3_), ammonia solution (NH_4_OH), sodium sulfate anhydrous (Na_2_SO_4_), methanol (CH_3_OH), and ethanol (C_2_H_6_O) were all analytical grade and purchased from Sinopharm Chemical Reagent Co., Ltd, China. All chemicals were used as received without further purification.

N-doped TiO_2_ was prepared using a typical hydrothermal method similar to our previous work [19]. A mixture of 1.5 g P25 powder and 70 ml of 10 M NaOH solution was heated at 150°C for 48 h in a Teflon-lined autoclave. After cooling, the precipitate was neutralized thoroughly by washing with a 0.1 M HNO_3_ solution and distilled water. Then, the precipitate was aged in a 0.5 M HNO_3_ solution and placed in a 0.5 M NH_4_OH solution for 24 h, respectively. The resulting powder was dried at 80°C for 8 h to obtain N-doped titanium nanotubes (NTNT). Yellow colored N-doped TiO_2_ was finally obtained after being calcined at 400°C for 1 h in air and designated as N-TiO_2_. Titanium nanotubes (TNT) and TiO_2_ samples for comparison were prepared in the same method as above without the steps of aging and ammonia impregnation.

The Au loading process was performed using a photoreduction method as described previously [20]. First, 0.3 g of N-TiO_2_ or TiO_2_ was dispersed into the mixture of 60 mL deionized water and 15 mL methanol, then a 628 µL solution of 10 mg/mL AuCl_3_•HCl•4H_2_O was added as the gold precursor (1 wt% or 0.41 at% Au loaded on the base material). The pH of the suspensions solution was adjusted to ca. 7.0 using 0.1 M NaOH, and the resulting mixture was dispersed by ultrasound for 15 min. The reaction system was magnetic stirred using a magnetic stirrer and ventilated under a continuous flow of Ar gas to remove the oxygen from the reactor. Four UV lamps (4 W, 254 nm, Xuanfeng, China) were used to irradiate the slurry for 2 h with an irradiation intensity of ca. 8 mW/cm^2^. Then, the precipitate was filtered and washed with distilled water three times. Finally, the solid was dried at 60°C overnight under air to obtain Au-modified N-doped TiO_2_ or Au-loaded TiO_2_ and designated Au/N-TiO_2_ or Au/TiO_2_, respectively.

### Material characterization

The crystal phases of the samples were analyzed by X-ray diffraction (XRD) patterns (X'Pert Pro, PANalytical, Holland) using Cu K*α* radiation (λ = 1.5418 Å) at 40 kV and 150 mA.

The morphology and nanostructure of samples were examined by a transmission electron microscopy (TEM, FEI, USA) and a high resolution TEM (HRTEM).

The surface properties of Au/N-TiO_2_ were investigated using X-ray photoelectron spectroscopy (XPS, Thermo ESCALAB 250, USA). Al K*α* radiation (h*ν* = 1486.6 eV) was used as the X-ray source. All bonding energies were calibrated to the C 1s internal standard.

The light absorption properties of the samples were analyzed by UV–vis diffuse reflectance spectra (DRS) using a UV–vis spectrophotometer (TU-1901, Pgeneral, China) equipped with an integrating sphere assembly.

The photoluminescence spectra (PL) were measured with a Fluorolog-3-Tau spectrophotometer using a UV lamp of 254 nm as the excitation source.

The incident visible and UV light intensities were detected using a radiometer (FZ-A, Handy, China) and a UV light meter (ST-512, Sentry, China), respectively.

### Photoelectrochemical measurements

Photoelectrochemical measurements were performed using an electrochemical workstation (CH Instruments 650D, Shanghai, China) with a standard three-electrode quartz cell, as described in our previous work [21]. Two UV lamps (4 W, 254 nm, Xuanfeng, China) and a 300 W xenon (Xe) lamp (CEL-HXUV300, CeAulight, China) filtered with VisREF (350–780 nm) and UVIRCUT400 (400–780 nm) were used as UV (ca. 2 mW/cm^2^) and visible light (ca. 20 mW/cm^2^) sources, respectively. A solution of 0.1 M Na_2_SO_4_ and absolute ethanol with a volume ratio of 4∶1 was used as electrolytes and degassed under nitrogen gas for 15 min prior to electrochemical measurements. Photocurrent densities vs. time curves were obtained at zero bias voltage irradiated with UV (*λ* = 254 nm) or visible light (λ>400 nm) for 20 s. Electrochemical impedance spectroscopy (EIS) was obtained in the frequency range from 1 Hz to 1 MHz at an amplitude of 5 mV, and the applied bias voltage was set at open-circuit voltage.

### Photocatalytic activity

Photocatalytic hydrogen production experiments were performed in a top-irradiation jacketed quartz photoreactor in which the temperature was maintained using flowing water in the jacket around the reactor. In a typical reaction, 50 mg of the photocatalyst was dispersed by magnetic stirring in a 50 mL of 30% methanol/water aqueous solution. Before illumination, high-purity Ar gas was bubbled for 1 h to completely remove residual oxygen. A 300 W xenon (Xe) lamp (CEL-HXUV300, CeAulight, China) filtered with VisREF (350–780 nm) and three UV lamps (4 W, 254 nm, Xuanfeng, China) were used as the UV–vis and UV light sources, respectively. The resulting gas was periodically detected using a gas chromatograph (Fuli 9790, China) equipped with a thermal conductivity detector at intervals of 1 h with Ar as the carrier gas.

## Results and Discussion

### Structure and morphology

**Figure 1:**
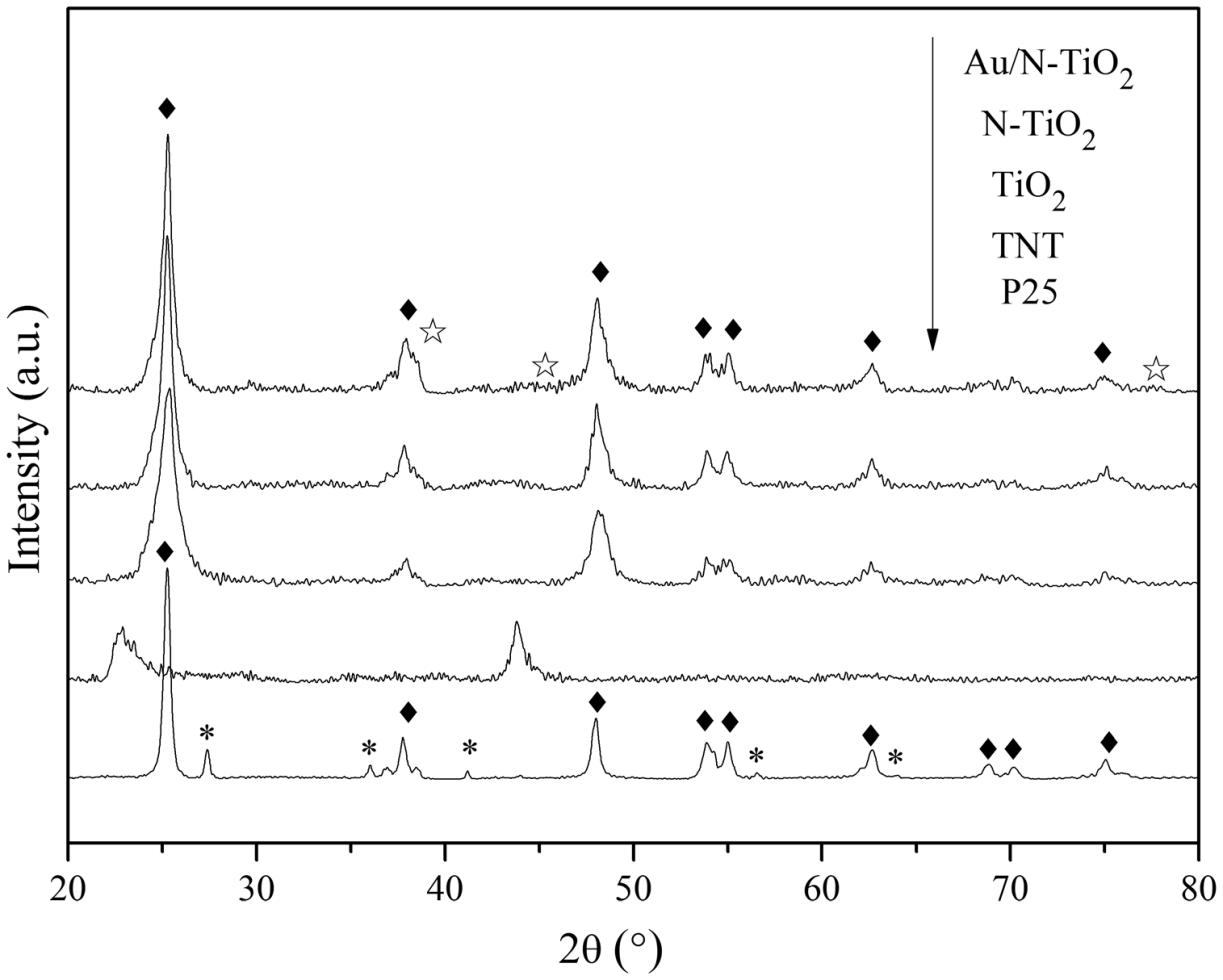
XRD patterns of P25, TNT, TiO_2_, N-TiO_2_ and Au/N-TiO_2_. Asterisk, rhombus, and open star denote rutile, anatase, and Au, respectively.

Figure 1 shows the XRD patterns of P25, TNT, TiO_2_, N-TiO_2_, and Au/N-TiO_2_. It can be observed that pure P25 exhibits diffraction peaks at 25.26°, 37.78°, 48.00°, 53.79°, 55.02°, 62.63°, 68.79°, 70.38°, and 75.10° indexed to the (101), (004), (200), (105), (211), (204), (116), (220), and (215) crystal planes of anatase TiO_2_ (JCPDS 21–1272), respectively. It also shows diffraction peaks at 27.39°, 36.02°, 41.22°, 56.61° and 64.01°, corresponding to the (110), (101), (111), (220), and (310) planes of rutile TiO_2_ (JCPDS 21–1276), respectively. After hydrothermal treatment, the as-prepared TNT does not show peaks corresponding to anatase, rutile, brookite, or the mixtures of them. According to the literature [22], the crystal structure of TNT can be designated as a structural variant of H_2_Ti_3_O_7_. The XRD patterns of TiO_2_, N-TiO_2_, and Au/N-TiO_2_ show the same peaks corresponding to anatase, indicating that H_2_Ti_3_O_7_ completely converted to anatase TiO_2_ during calcination. The results also demonstrate that the implanted nitrogen and deposited Au have almost no effect on the crystal orientations of TiO_2_. New peaks at 38.21°, 44.38°, and 77.56°, corresponding to (111), (200), and (311) planes of polycrystalline Au (JCPDS 04–0784), appear in the XRD pattern of Au/N-TiO_2_, confirming the existence of Au. These peaks are not particularly obvious, which could be attributed to the low concentration of Au.

**Figure 2:**
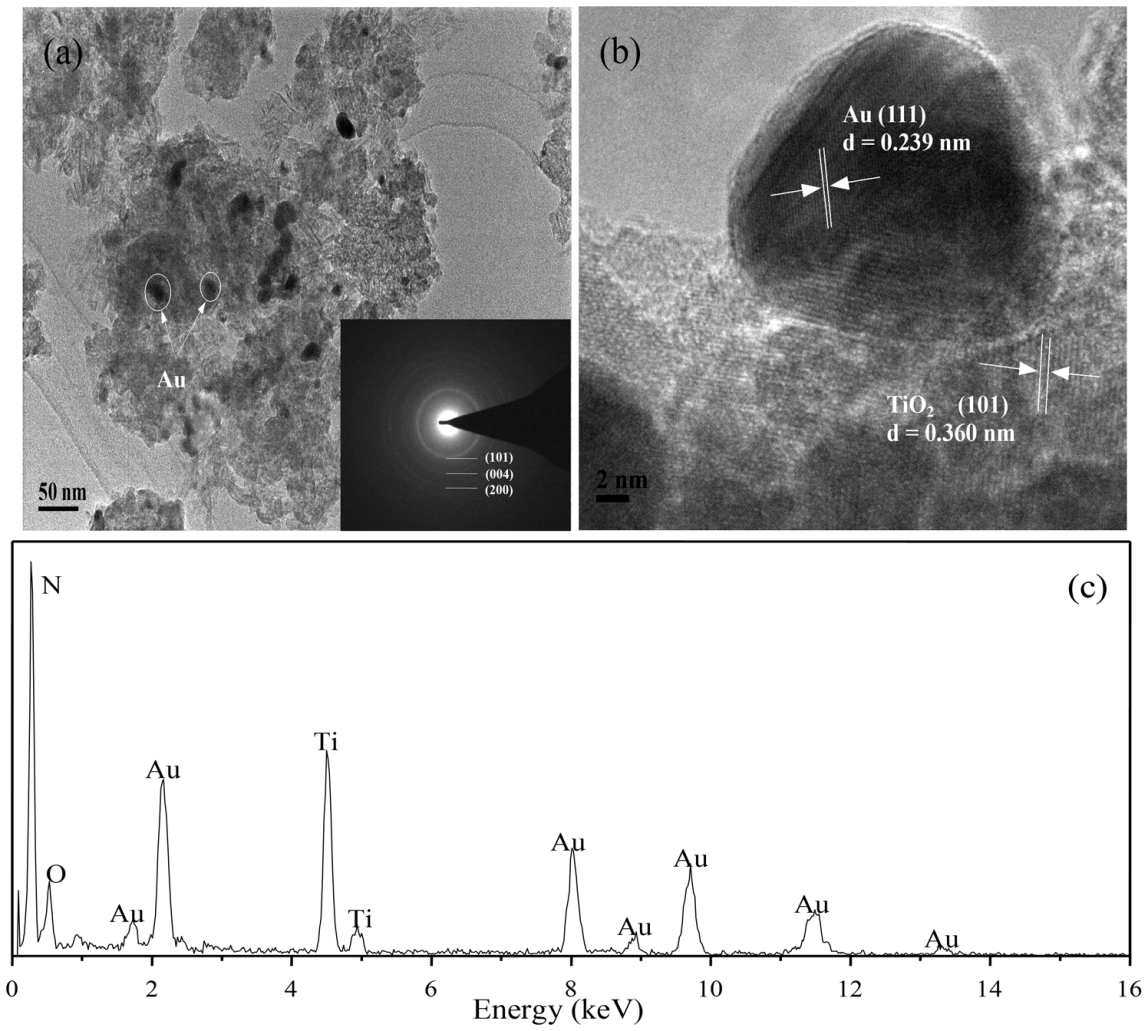
(a) TEM image of as synthesized Au/N-TiO_2_, inset in the lower right corner is the SAED pattern of anatase TiO_2_ and (b) HRTEM image and (c) EDX spectrum of Au/N-TiO_2_.

TEM images of Au/N-TiO_2_ are displayed in Figure 2a. After calcination, the Au/N-TiO_2_ photocatalysts turn into nano-bulk, and the Au particles attached onto the surface of N-TiO_2_ are approximately 20–30 nm. The SAED pattern in the inset of Figure 2a shows rings that correspond to anatase TiO_2_ (101), (004), and (200). Figure 2b shows a high-resolution TEM (HRTEM) image of a representative Au nanoparticle attached to N-TiO_2_; two distinct lattice spacings are observed, which match the interplanar spacings of anatase TiO_2_ and Au, respectively. The measured distance between TiO_2_ lattice planes is 0.360 nm, which corresponds to anatase TiO_2_ (101), and the lattice fringes corresponding to the (111) plane of Au are 0.239 nm. The EDS spectrum of Au/N-TiO_2_ indicates the presence of N and Au (Figure 2c).

### Surface analysis

**Figure 3:**
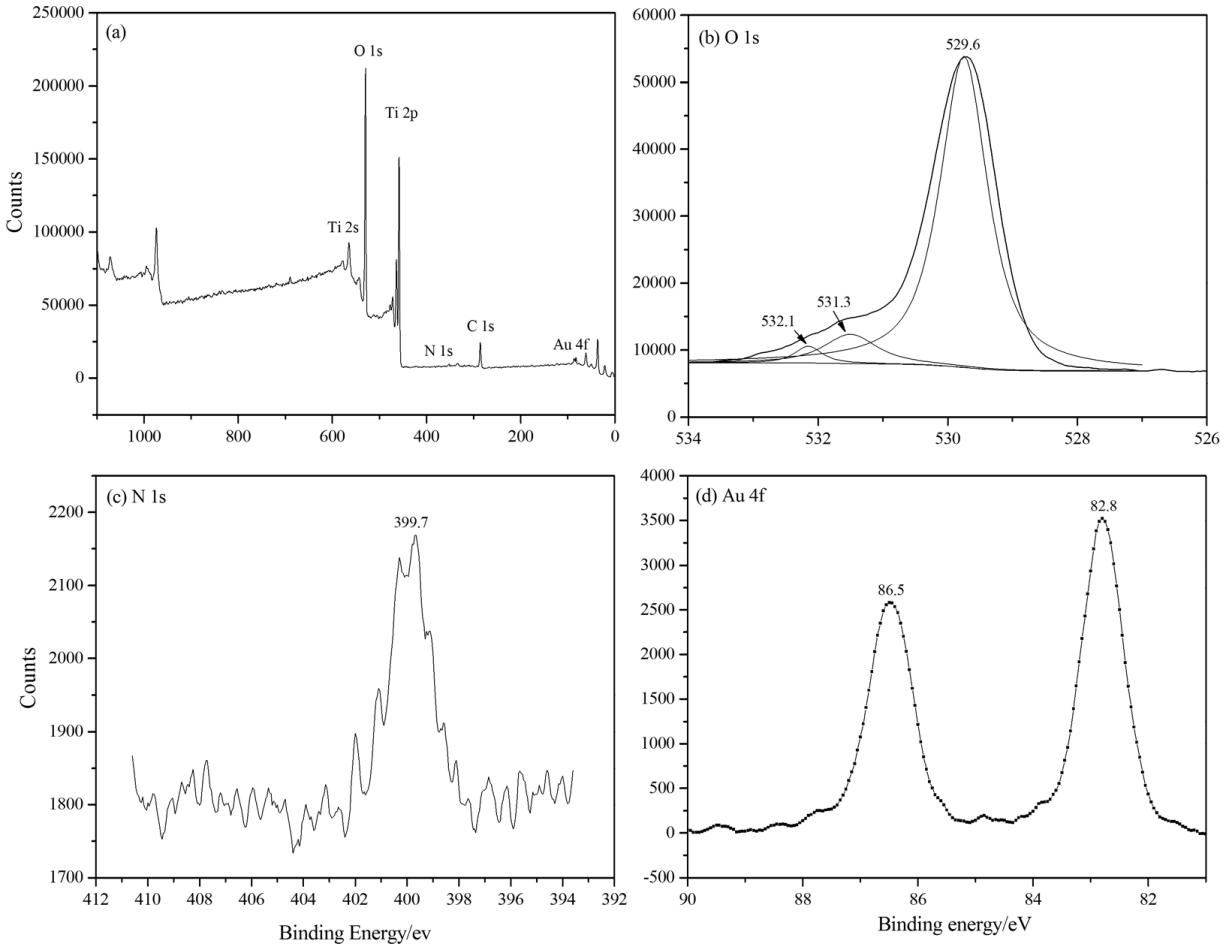
XPS spectra of (a) Au/N-TiO_2_ and core level spectra of (b) O 1s, (c) N 1s, and (d) Au 4f.

XPS was used to determine the chemical state of Au/N-TiO_2_ (Figure 3). Figure 3a shows the entire XPS survey of Au/N-TiO_2_. The presence of the C 1s peak located at a binding energy (BE) of 286.0 eV is attributed to the adhesive tape used in the XPS measurement. Figure 3b shows the high-resolution XPS spectra for the O 1s region of Au/N-TiO_2_. Three peaks at 529.6, 531.3, and 532.1 eV can be observed, indicating three different O state types. According to the literature [23], [24], peaks centered at 529.6 and 531.3 eV can be assigned to oxygen in Ti–O–Ti and Ti–O–N bonds in the lattice, respectively. The third peak at 532.1 eV can be attributed to the Ti–O–H bonds resulting from chemisorbed water [25]. There are two types of N species in TiO_2_ lattice, including interstitial N (Ni^•^) and substitutional N (Ns^•^). Ni^•^ proved to be more favorable from the viewpoint of energy based on DFT calculations [26]. N 1s XPS spectra are shown in Figure 3c. After nitrogen doping, an N 1s peak with a core level BE of 399.7 eV is observed, as also reported in the literature [27], [28]. The result proves that the doped N species are in interstitial positions bound directly to lattice oxygen, which is consistent with the theoretical calculations, and the linkages to nitrogen atoms may be in the state of Ti–O–N or Ti–N–O [29]. Figure 3d shows the XPS Au 4f spectra of the Au/N-TiO_2_ samples. Double peaks for gold nanoparticles are found at 82.8 and 86.5 eV, corresponding to Au 4f7/2 and Au 4f5/2, indicating a shift of the Au 4f peaks toward lower binding energies compared with the peaks located at 83.3 and 87.2 eV [30], [31], respectively, which is most likely due to the intimate contact between the Au and the N-TiO_2_ substrate, leading to a change in the electronic properties [18]. The amount of Au and N measured by XPS were 0.27 at% and 0.18 at%, respectively.

### Optical properties

**Figure 4:**
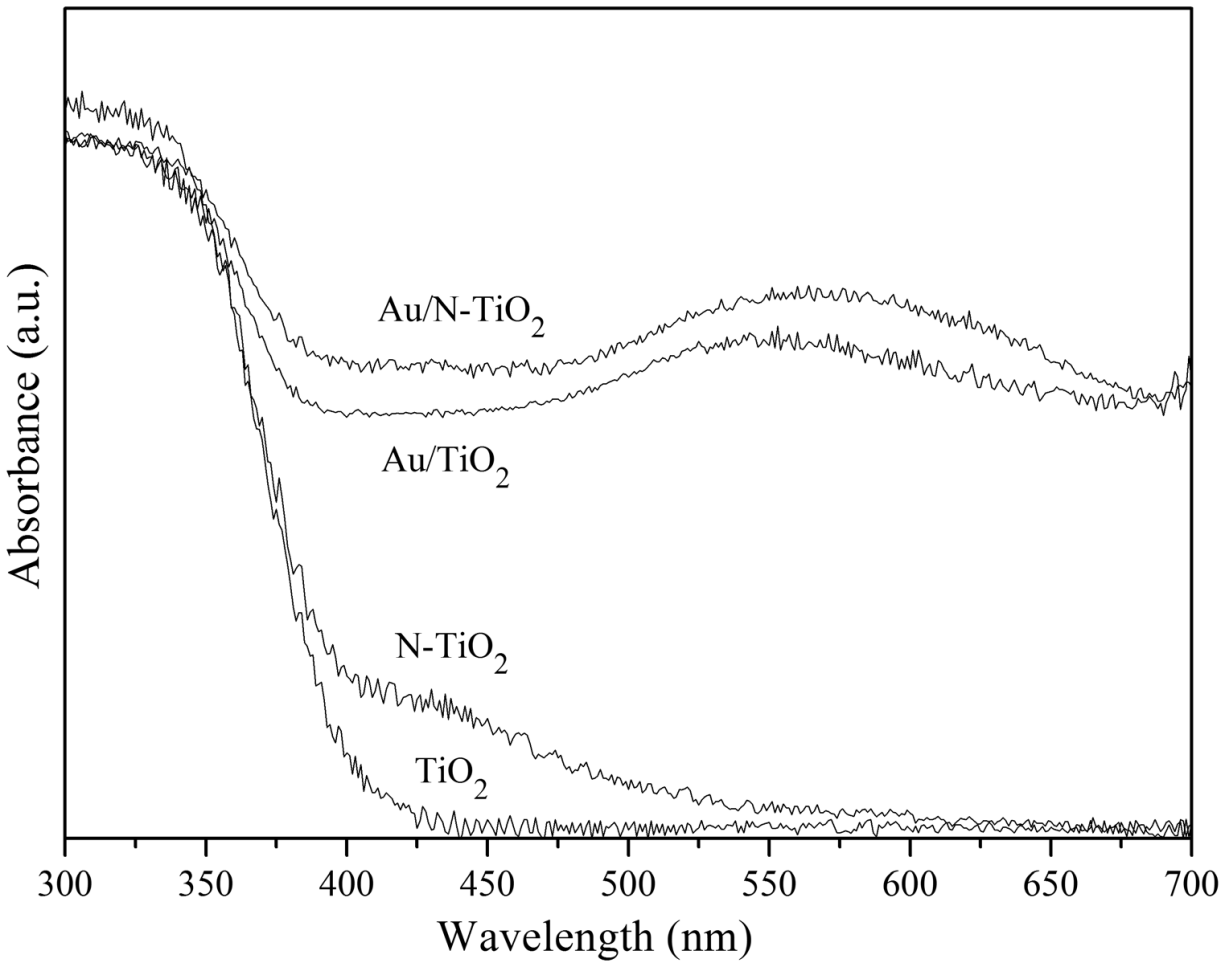
UV–vis diffuse reflectance spectra of TiO_2_, Au/TiO_2_, N-TiO_2_, and Au/N-TiO_2_.

Figure 4 shows the DRS of TiO_2_, Au/TiO_2_, N-TiO_2_, and Au/N-TiO_2_. It can be observed that the absorption spectrum of TiO_2_ is cut off at 400 nm, while N-TiO_2_ sample shows significant absorption in the visible region between 400 and 550 nm. It was reported that single-atom nitrogen impurities (including Ni^•^ and Ns^•^) can form either diamagnetic (N_b_^−^) or paramagnetic (N_b_^•^) bulk centers, and both of them can give rise to localized states in the band gap of the oxide [32]. Under irradiation, a reversible electron transfer between charged diamagnetic N_b_^−^ and neutral paramagnetic N_b_^•^ can form, simultaneously resulting in the excitation of electrons into the conduction band; therefore, they play an essential role in the absorption of visible light [33]. Oxygen vacancies induced by nitrogen doping in TiO_2_ can act as color centers and can also contribute to the visible light absorption [8]. In addition, after loading with Au, another absorbance band at approximately 550 nm can be found and is attributed to the surface plasmonic resonance (SPR) effect of the Au nanoparticles [34], [35]. Metal nanoparticles, such as Au, Cu, and Ag, are able to absorb and scatter photons in the visible region. When the particles are illuminated, the free conduction band electrons will oscillate under the force exerted by the electromagnetic field of the light, resulting in a strong-field enhancement of the local electromagnetic field and the appearance of plasmon absorption bands [36]. This near-field optical enhancement is hypothesized to increase the electron-hole pair generation rate and to enhance the photocatalytic activity accordingly [37], [38].

**Figure 5:**
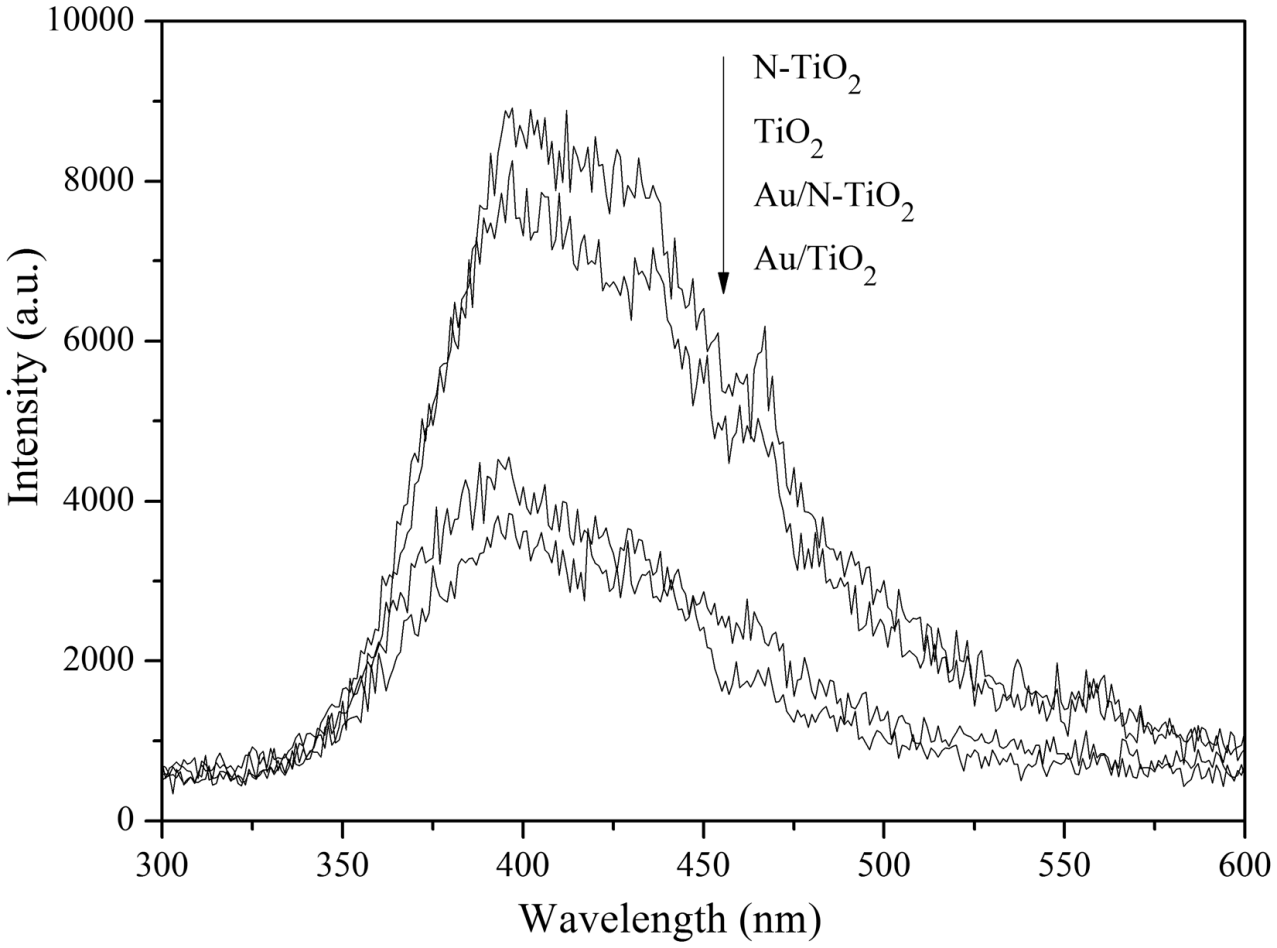
PL emission spectra of TiO_2_, Au/TiO_2_, N-TiO_2_, and Au/N-TiO_2_ under the irradiation of 254 nm. nm.

PL emission spectra were used to measure the altered electronic properties of the as-prepared photocatalysts, including the immigration, transfer, and recombination processes of the charge carriers [39], [40]. Figure 5 demonstrates the PL spectra of TiO_2_, Au/TiO_2_, N-TiO_2_, and Au/N-TiO_2_. A broad signal in the excitonic PL spectra from 350 to 500 nm can be observed in these samples, resulting from the transition of excitons, such as photoinduced electrons trapped by oxygen vacancies and defects and the recombination of photo-induced electrons and holes. It can be observed that the PL intensity of N-TiO_2_ and Au/N-TiO_2_ are higher than that of TiO_2_ and Au/TiO_2_, respectively, which can be attributed to the implanted N species. It was reported that the localized N doping level, oxygen vacancies, and other defect states, such as the Ti 3d state, can trap photogenerated electrons, thus inducing a high recombination rate [41]. The PL intensities of the Au-loaded samples (Au/TiO_2_ and Au/N-TiO_2_) are much lower than those of the samples without Au (TiO_2_ and N-TiO_2_), implying a lower recombination rate for the electrons and holes, which is mainly dependent on the formation of a Schottky barrier at the interface between Au/TiO_2_ and Au/N-TiO_2_; this barrier can rapidly enhance the transfer of photo-induced electrons and simultaneously retard the recombination of electrons and holes [42]. The Fermi level of Au is lower than that of TiO_2_. When these two materials are electrically connected, photogenerated electrons in TiO_2_ can readily be transferred to the Au nanoparticles and form a new quasi-Fermi level (E^'^_F_) [43].

### Photocatalytic activity

**Figure 6:**
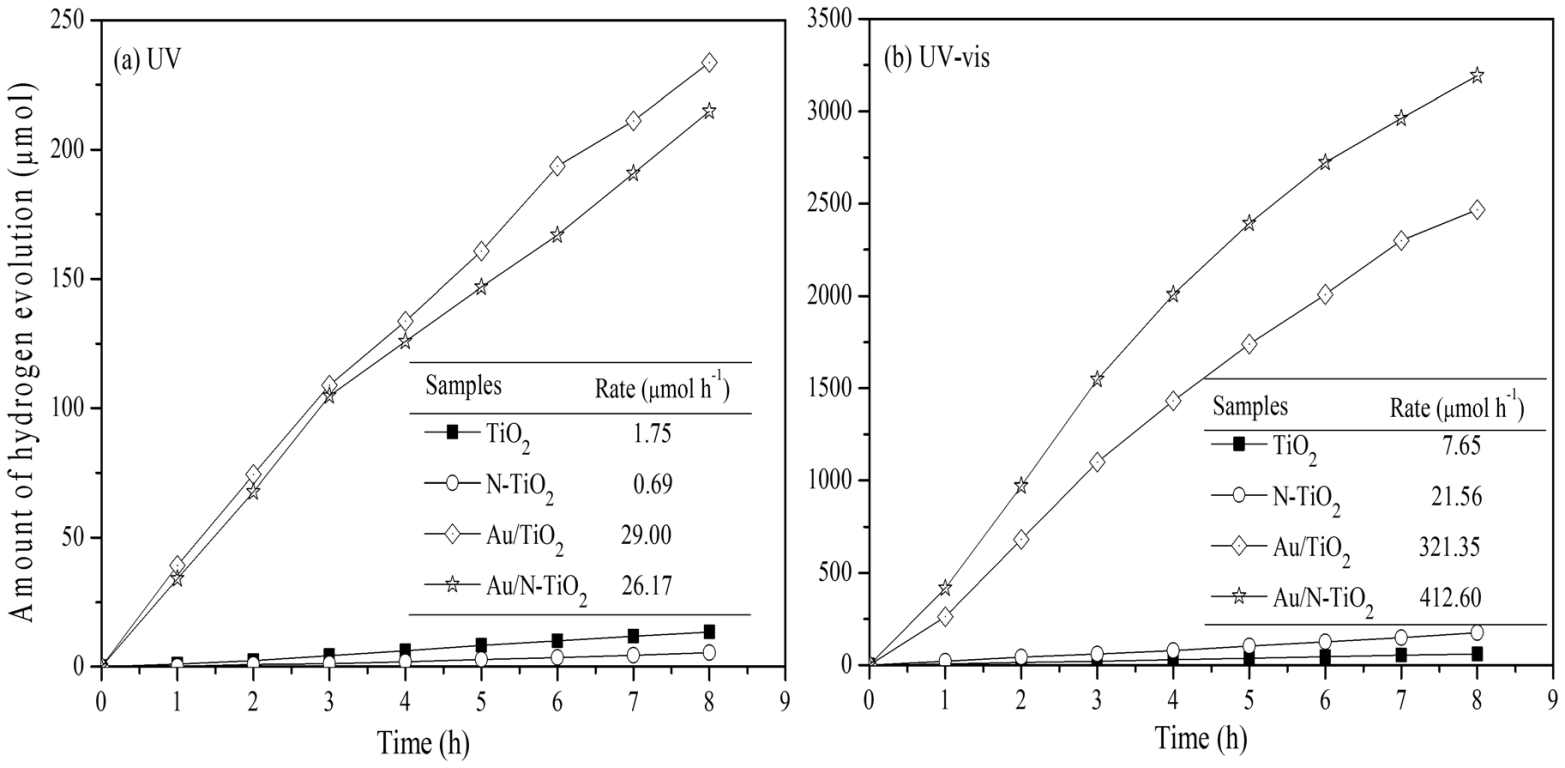
Photocatalytic activity for water splitting under the irradiation of (a) UV and (b) UV–vis light.

The H_2_ evolution from photocatalytic water splitting reaction under irradiation of UV and UV–vis light was used to evaluate the photocatalytic performance of TiO_2_, N-TiO_2_, Au/TiO_2_, and Au/N-TiO_2_, as shown in Figure 6. Under the irradiation of UV light (Figure 6a), the N-TiO_2_ and Au/N-TiO_2_ samples show lower H_2_ evolution rates than their corresponding samples without nitrogen doping (0.69 and 1.75 µmol·h^−1^ for N-TiO_2_ and TiO_2_, respectively; 26.17 and 29.00 µmol·h^−1^ for Au/N-TiO_2_ and Au/TiO_2_, respectively), confirming that the N doping has an acceleration on photogenerated electrons and holes recombination. After Au loading on pure TiO_2_ and N-TiO_2_, the H_2_ production are greatly enhanced due to the effective segregation of photo-induced electrons and holes resulting from the Schottky barrier at the interface of Au/TiO_2_ and Au/N-TiO_2_ [44], which is consistent with the result of PL. Under the irradiation of UV–vis light (Figure 6b), however, the N-TiO_2_ and Au/N-TiO_2_ samples show higher H_2_ evolution rates than their corresponding nitrogen-free samples (TiO_2_ and Au/TiO_2_). The H_2_ evolution rates increase from 7.65 (TiO_2_) and 321.35 (Au/TiO_2_) µmol·h^−1^ to 21.56 (N-TiO_2_) and 412.60 (Au/N-TiO_2_) µmol·h^−1^, respectively. For N-TiO_2_, the enhancement of photocatalytic activity can be attributed to the doping of N, which can induce a visible light response in the TiO_2_ original photocatalyst. The Au/N-TiO_2_ sample has the highest UV-vis light hydrogen evolution rate because both the N doping and the SPR effect from the Au particles can induce a visible light response. That is, the cooperative effect of the N dopant and the Au particles results in an enhancement of the visible light photocatalytic activity for H_2_ evolution, thereby greatly enhancing the utilization of sunlight.

**Figure 7:**
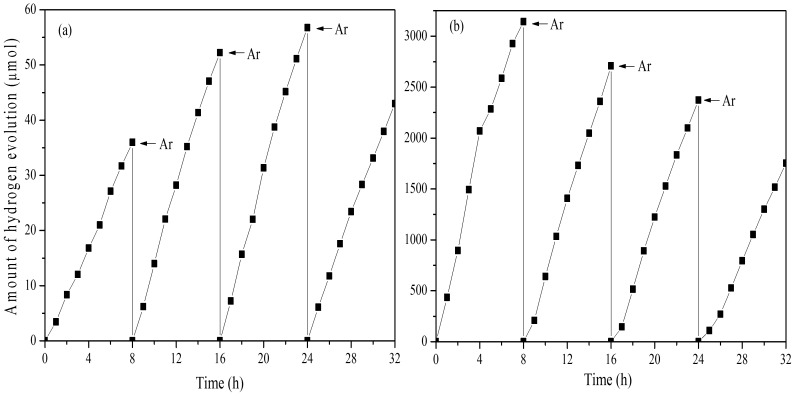
The repeated hydrogen evolution tests over Au/N-TiO_2_ under UV-vis light in (a) pure water and (b) methanol/water solution with purging of Ar in every 8 h. h.

The repeated hydrogen evolution tests over Au/N-TiO_2_ under UV-vis light in pure water and in methanol/water solution were performed by purging of Ar in every 8 h. Fig 7a shows that the photocatalyst is able to split water for hydrogen evolution without adding methanol and the stability of this photocatalyst is good. This, in turn, indicates that methanol can greatly enhance the hydrogen production rate as a sacrificial agent. The stability of Au/N-TiO_2_ photocatalyst in methanol/water solution is shown in Fig 7b, and as the increase of recycling times, the photocatalytic activity decrease slightly probably due to the loss of methanol in every recycling experiment.

### Photoelectrochemical characterizations

**Figure 8:**
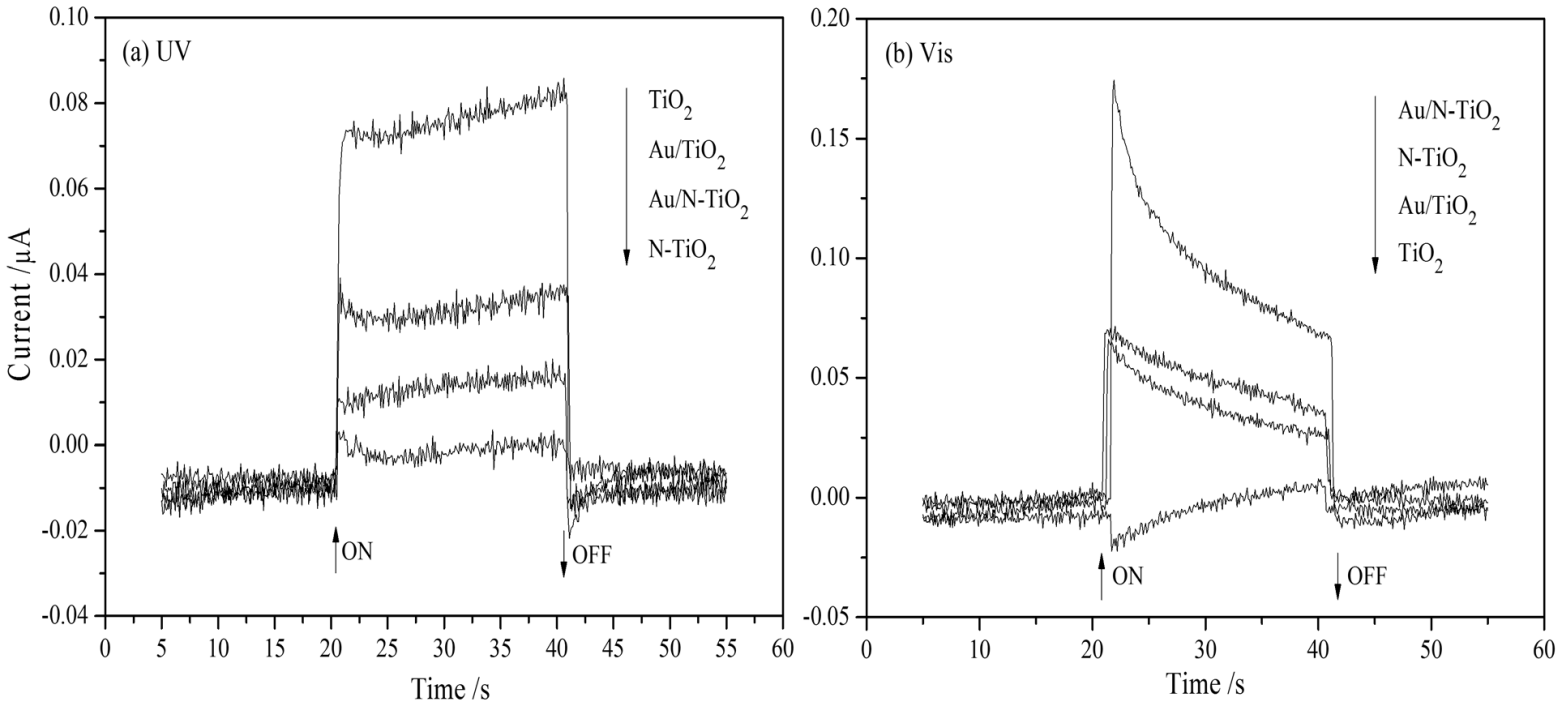
Photocurrents of TiO_2_, N-TiO_2_, Au/TiO_2_, and Au/N-TiO_2_ electrodes at zero bias voltage irradiated with (a) UV (*λ* = 254 nm nm) and (b) visible light (λ>400 nm nm) for 20 s. s.

To further investigate the photoelectric properties of the as-prepared samples, photoelectrochemical measurements were performed. The transient photocurrents of TiO_2_, N-TiO_2_, Au/TiO_2_, and Au/N-TiO_2_ under the irradiation of UV and visible light for 20 s are shown in Figure 8. Under the illumination of UV light (Figure 8a), the photocurrent responses of as-prepared samples follow the order TiO_2_ > Au/TiO_2_ > Au/N-TiO_2_ > N-TiO_2_. While the currents of photocatalytic activity are as follows: Au/TiO_2_ > Au/N-TiO_2_ > TiO_2_ > N-TiO_2_. This small inconsistency can be attributed to two aspects: 1) the deposited gold nanoparticles decrease the number of photons reaching the TiO_2_ and N-TiO_2_ surface and reduce the surface area in contact with the electrolytes [37] and 2) electrons hopping across the particle boundaries are trapped on the surfaces of the Au/TiO_2_ and Au/N-TiO_2_ electrodes, decreasing the efficiency of the photocurrent collection at the ITO base [45]. Under visible light irradiation (Figure 8b), TiO_2_ shows a lower photocurrent than N-TiO_2_, Au/TiO_2_, or Au/N-TiO_2_. For N-TiO_2_, the photocurrent response is attributed to the extension of the photonic absorption due to N doping. For Au/TiO_2_, the measured photocurrent result proves the SPR effect due to the modified Au particles. The highest photocurrent response from Au/N-TiO_2_ suggests cooperation between the visible light response of the N dopant and the SPR effect of the Au particles, which is consistent with the results in the Photocatalytic Activity section.

**Figure 9:**
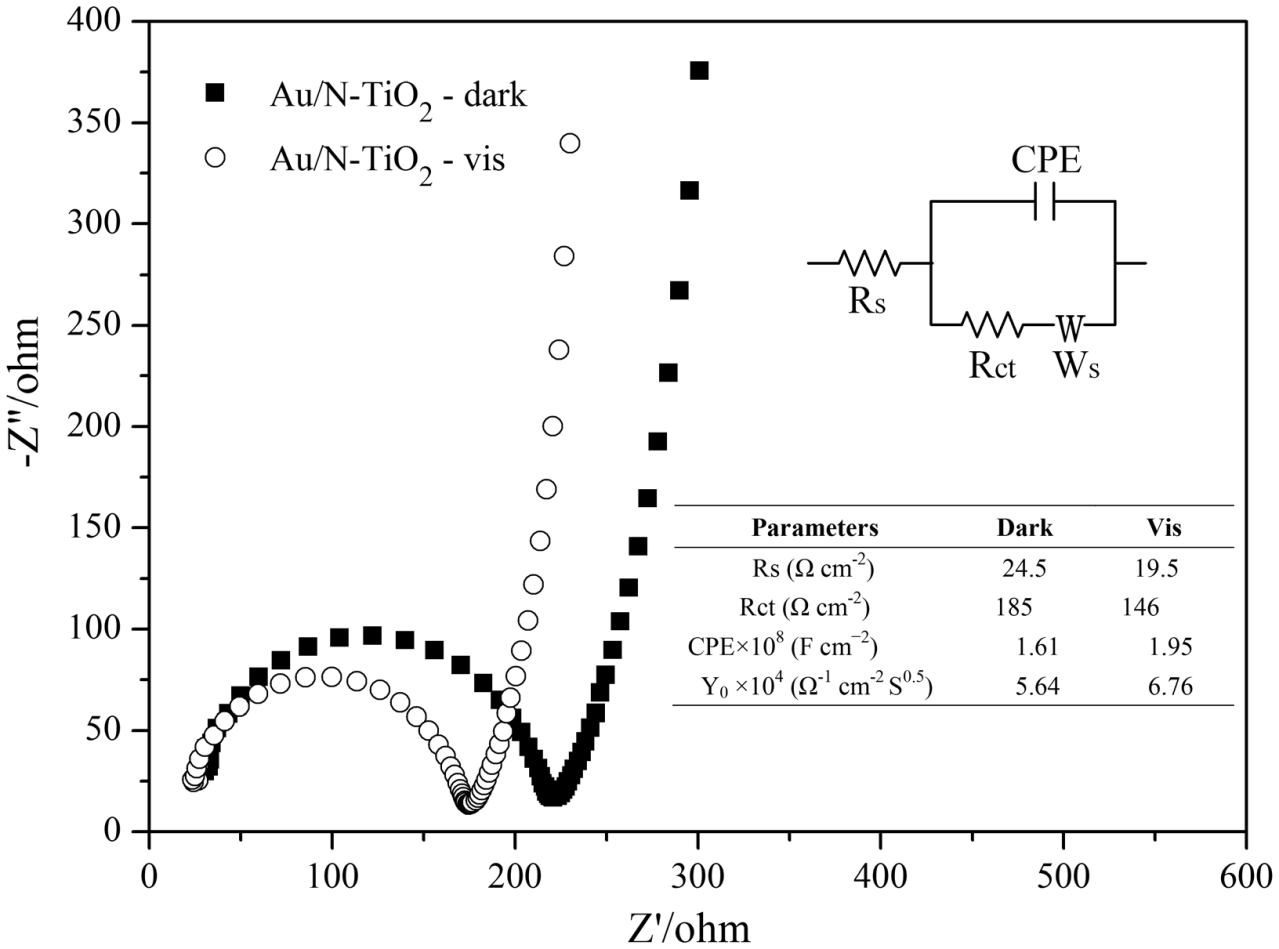
EIS Nyquist plots for Au/N-TiO_2_ in dark and under the irradiation of visible light. Inset is the suggested equivalent circuit and the fitting results for Au/N-TiO_2_. Rs and Rct are the electrolyte and electron-transfer resistance, respectively. CPE is the constant phase element, which also represents the double layer capacitance. Ws is the Warburg impedance. Y_0_ is the value of admittance and expresses a reciprocal relationship to the Warburg coefficient, which is able to predict the Warburg impedance and diffusion coefficient.

EIS for Au/N-TiO_2_ was obtained to further investigate the cooperation between N doping and Au loading on the charge separation efficiency under visible light irradiation (Figure 9). The Nyquist plots show a pronounced semicircle at the high frequencies and a straight sloping line at low frequencies in both the dark and the irradiated cases. The diameters of the semicircles correspond to the charge-transfer resistance at the electrode interface, whereas the straight sloping line is related to the diffusion process of the reactive species [46]. The inset in Figure 9 is the proposed equivalent circuit and the fitting results for Au/N-TiO_2_. Rs is the electrolyte resistance and also includes the contact and charge transfer resistances at the counter electrode/electrolyte [47]. Rct is the electron-transfer resistance. CPE is the constant phase element, which also represents the double layer capacitance. Ws is the Warburg impedance, which is related to the diffusion of the reactive species at the surface of the electrodes and is illustrated by the straight line in the Nyquist plots. The contribution of the visible light response for doped N and loaded Au is identified by the decreasing of Rct and the increasing of double layer capacitance under illumination because TiO_2_ is not photoactive under visible light. As observed, the value of Rct for the Au/N-TiO_2_ electrode decreases from 185 Ω·cm^−2^ in the dark to 146 Ω·cm^−2^ under visible light illumination, while the value of CPE increases from 1.61×10^−8^ F·cm^−2^ in the dark to 1.95×10^−8^ F·cm^−2^ under visible light illumination, manifesting as a faster charge transfer under visible light illumination due to the N doping and Au loading [48].

**Figure 10:**
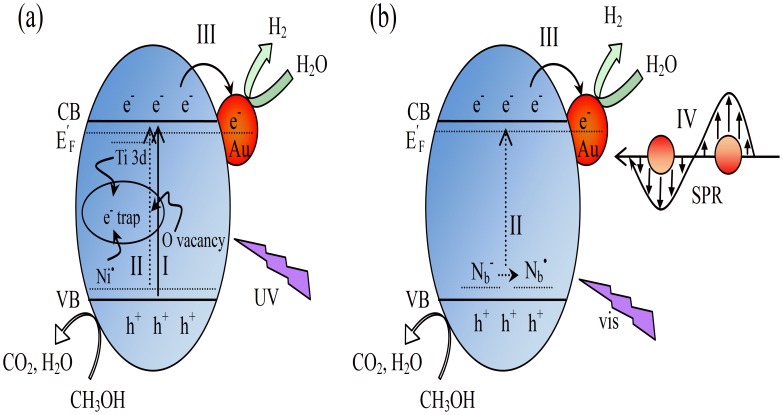
Schematic illustration of Au/N-TiO_2_ for water splitting under the irradiation of (a) UV and (b) visible light. Pathway I denotes the generation of charge carriers in TiO_2_. Pathway II represents the reversible electron transfer between charged diamagnetic N_b_^−^ and neutral paramagnetic N_b_^•^, and the excitation of electrons into the conduction band. Pathway III shows the acceleration of photo-induced electrons transfer by Au loading. Pathway IV denotes the SPR effect of loaded Au nanoparticles.

Figure 10 depicts sketches of the main charge-transfer process of Au/N-TiO_2_ for water splitting. Under UV irradiation (Figure 10a), charges are mainly generated in the TiO_2_. The N dopant, oxygen vacancies and Ti 3d state can trap photogenerated electrons, delaying the separation of the electrons and holes. The Schottky barrier at the Au/N-TiO_2_ interface can rapidly enhance the transfer of photoinduced electrons to Au nanoparticles and boost the photocatalytic activity for hydrogen production. Under visible light irradiation (Figure 10b), both the implanted N and the loaded Au contribute to the generation of electrons. Diamagnetic N_b_^−^ can transform into neutral paramagnetic N_b_^•^ and simultaneously excite electrons from the N states into the conduction band. The strong local electromagnetic field will increase the electron-hole pair generation rate in Au and enhance the visible light photocatalytic activity accordingly. The Au nanoparticles also play an important role in increasing the transfer of electrons under visible light irradiation.

## Conclusions

Au/N-TiO_2_ photocatalyst was prepared via hydrothermal synthesis and ammonia impregnation and followed by a typical photoreduction method. XRD characterization described the change in the crystal structure from P25 to Au/N-TiO_2_. TEM and XPS indicated that metallic Au can be loaded as Au^0^ on the surface of the N-TiO_2_ photocatalyst, and the nitrogen species were in the form of interstitial nitrogen. The results from the DRS, PL, and photoelectrochemical characterization indicated an extension of the light absorption into the visible region after Au loading and N doping, and the electron-hole recombination rate was effectively inhibited by the Au particles. Under UV light irradiation, the N-TiO_2_ and Au/N-TiO_2_ samples showed lower H_2_ evolution than the corresponding samples without nitrogen (TiO_2_ and Au/TiO_2_). Under UV–vis light irradiation, the N-TiO_2_ and Au/N-TiO_2_ samples showed more H_2_ evolution than the corresponding nitrogen-free samples (TiO_2_ and Au/TiO_2_). This finding indicated a synergic effect between the doped N and the Au in the enhancement of the visible light photocatalytic activity for H_2_ evolution. Through N doping and Au loading, the as-prepared photocatalysts successfully increased the utilization of sunlight and enhanced the photocatalytic activity for H_2_ evolution.

## Supporting Information

File S1 (DOCX)

## References

[1] Jia T, Kolpin A, Ma C, Chan RCT, Kwok WM, et al (2014) A graphene dispersed CdS-MoS_2_ nanocrystal ensemble for cooperative photocatalytic hydrogen production from water. Chem Commun 50: 1185–1188. 10.1039/c3cc47301e 24326768

[2] Yang G, Yan W, Zhang Q, Shen S, Ding S (2013) One-dimensional CdS/ZnO core/shell nanofibers via single-spinneret electrospinning: tunable morphology and efficient photocatalytic hydrogen production. Nanoscale 5: 12432–12439. 24166349 10.1039/c3nr03462c

[3] Wang D, Hisatomi T, Takata T, Pan C, Katayama M, et al (2013) Core/shell photocatalyst with spatially separated co-catalysts for efficient reduction and oxidation of water. Angew Chem Int Edit 52: 11252–11256. 10.1002/anie.201303693 23946215

[4] Zheng Z, Zhao J, Yuan Y, Liu H, Yang D, et al (2013) Tuning the surface structure of nitrogen-doped TiO_2_ nanofibres–an effective method to enhance photocatalytic activities of visible-light-driven green synthesis and degradation. Chem-Eur J 19: 5731–5741. 23463719 10.1002/chem.201203961

[5] Xiang Q, Yu J, Wang W, Jaroniec M (2011) Nitrogen self-doped nanosized TiO_2_ sheets with exposed {001} facets for enhanced visible-light photocatalytic activity. Chem Commun 47: 6906–6908. 10.1039/c1cc11740h 21556416

[6] Lin Z, Orlov A, Lambert RM, Payne MC (2005) New insights into the origin of visible light photocatalytic activity of nitrogen-doped and oxygen-deficient anatase TiO_2_ . J Phys Chem B 109: 20948–20952. 16853715 10.1021/jp053547e

[7] Zhang J, Wu Y, Xing M, Leghari SAK, Sajjad S (2010) Development of modified N doped TiO_2_ photocatalyst with metals, nonmetals and metal oxides. Energ Environ Sci 3: 715–726.

[8] Wang J, Tafen DN, Lewis JP, Hong Z, Manivannan A, et al (2009) Origin of photocatalytic activity of nitrogen-doped TiO_2_ nanobelts. J Am Chem Soc 131: 12290–12297. 19705915 10.1021/ja903781h

[9] Taboada E, Angurell I, Llorca J (2014) Dynamic photocatalytic hydrogen production from ethanol-water mixtures in an optical fiber honeycomb reactor loaded with Au/TiO_2_ . J Catal 309: 460–467.

[10] Kominami H, Yamamoto S, Imamura K, Tanaka A, Hashimoto K (2014) Photocatalytic chemoselective reduction of epoxides to alkenes along with formation of ketones in alcoholic suspensions of silver-loaded titanium (iv) oxide at room temperature without the use of reducing gases. Chem Commun 50: 4558–4560. 10.1039/c3cc49340g 24668000

[11] Ohno T, Higo T, Murakami N, Saito H, Zhang Q, et al (2014) Photocatalytic reduction of CO_2_ over exposed-crystal-face-controlled TiO_2_ nanorod having a brookite phase with co-catalyst loading. Appl Catal B-Environ 152: 309–316.

[12] Wang P, Huang B, Zhang X, Qin X, Dai Y, et al (2011) Highly efficient visible light plasmonic photocatalysts Ag@Ag (Cl, Br) and Ag@AgCl-AgI. ChemCatChem 3: 360–364.

[13] Zhou X, Liu G, Yu J, Fan W (2012) Surface plasmon resonance-mediated photocatalysis by noble metal-based composites under visible light. J Mater Chem 22: 21337–21354.

[14] Seh ZW, Liu S, Low M, Zhang SY, Liu Z, et al (2012) Janus Au-TiO_2_ photocatalysts with strong localization of plasmonic near-fields for efficient visible-light hydrogen generation. Adv Mater 24: 2310–2314. 22467121 10.1002/adma.201104241

[15] Li P, Wei Z, Wu T, Peng Q, Li Y (2011) Au-ZnO hybrid nanopyramids and their photocatalytic properties. J Am Chem Soc 133: 5660–5663. 21446650 10.1021/ja111102u

[16] Graciani J, Nambu A, Evans J, Rodriguez JA, Sanz JF (2008) Au↔N synergy and N-doping of metal oxide-based photocatalysts. J Am Chem Soc 130: 12056–12063. 18700756 10.1021/ja802861u

[17] Tian B, Li C, Gu F, Jiang H (2009) Synergetic effects of nitrogen doping and Au loading on enhancing the visible-light photocatalytic activity of nano-TiO_2_ . Catal Commun 10: 925–929.

[18] Wu Y, Liu H, Zhang J, Chen F (2009) Enhanced photocatalytic activity of nitrogen-doped titania deposited with gold. J Phys Chem C 113: 14689–14695.

[19] Dong F, Zhao W, Wu Z (2008) Characterization and photocatalytic activities of C, N and S co-doped TiO_2_ with 1D nanostructure prepared by the nano-confinement effect. Nanotechnology 19: 365607. 21828878 10.1088/0957-4484/19/36/365607

[20] Iliev V, Tomova D, Todorovska R, Oliver D, Petrov L, et al (2006) Photocatalytic properties of TiO_2_ modified with gold nanoparticles in the degradation of oxalic acid in aqueous solution. Appl Catal A-Gen 313: 115–121.

[21] Zhao W, Zhang J, Zhu X, Zhang M, Tang J, et al (2013) Enhanced nitrogen photofixation on Fe-doped TiO_2_ with highly exposed (101) facets in the presence of ethanol as scavenger. Appl Catal B-Environ 144: 468–477.

[22] Mao Y, Wong SS (2006) Size-and shape-dependent transformation of nanosized titanate into analogous anatase titania nanostructures. J Am Chem Soc 128: 8217–8226. 16787086 10.1021/ja0607483

[23] Erdem B, Hunsicker RA, Simmons GW, Sudol ED, Dimonie VL, et al (2001) XPS and FTIR surface characterization of TiO_2_ particles used in polymer encapsulation. Langmuir 17: 2664–2669.

[24] Wu D, Long M, Cai W, Chen C, Wu Y (2010) Low temperature hydrothermal synthesis of N-doped TiO_2_ photocatalyst with high visible-light activity. J Alloy Compd 502: 289–294.

[25] Sreethawong T, Laehsalee S, Chavadej S (2009) Use of Pt/N-doped mesoporous-assembled nanocrystalline TiO_2_ for photocatalytic H_2_ production under visible light irradiation. Catal Commun 10: 538–543.

[26] Di Valentin C, Finazzi E, Pacchioni G, Selloni A, Livraghi S, et al (2007) N-doped TiO_2_: theory and experiment. Chem Phys 339: 44–56.

[27] Jiang Z, Yang F, Luo N, Chu BT, Sun D, et al.. (2008) Solvothermal synthesis of N-doped TiO_2_ nanotubes for visible-light-responsive photocatalysis. Chem Commun: 6372–6374. 10.1039/b815430a 19048159

[28] Wang J, Zhu W, Zhang Y, Liu S (2007) An efficient two-step technique for nitrogen-doped titanium dioxide synthesizing: visible-light-induced photodecomposition of methylene blue. J Phys Chem C 111: 1010–1014.

[29] Li X, Fan T, Zhou H, Zhu B, Ding J, et al (2008) A facile way to synthesize biomorphic N-TiO_2_ incorporated with Au nanoparticles with narrow size distribution and high stability. Micropor Mesopor Mat 116: 478–484.

[30] Chen JJ, Wu JCS, Wu PC, Tsai DP (2011) Plasmonic photocatalyst for H_2_ evolution in photocatalytic water splitting. J Phys Chem C 115: 210–216.

[31] Epling WS, Hoflund GB, Weaver JF, Tsubota S, Haruta M (1996) Surface characterization study of Au/α-Fe_2_O_3_ and Au/Co_3_O_4_ low-temperature CO oxidation catalysts. J Phys Chem C 100: 9929–9934.

[32] Di Valentin C, Pacchioni G, Selloni A (2004) Origin of the different photoactivity of N-doped anatase and rutile TiO_2_ . Phys Rev B 70: 085116.

[33] Livraghi S, Paganini MC, Giamello E, Selloni A, Di Valentin C, et al (2006) Origin of photoactivity of nitrogen-doped titanium dioxide under visible light. J Am Chem Soc 128: 15666–15671. 17147376 10.1021/ja064164c

[34] Tanaka A, Ogino A, Iwaki M, Hashimoto K, Ohnuma A, et al (2012) Gold-titanium (iv) oxide plasmonic photocatalysts prepared by a colloid-photodeposition method: correlation between physical properties and photocatalytic activities. Langmuir 28: 13105–13111. 22900610 10.1021/la301944b

[35] DeSario PA, Pietron JJ, DeVantier DE, Brintlinger TH, Stroud RM, et al (2013) Plasmonic enhancement of visible-light water splitting with Au-TiO_2_ composite aerogels. Nanoscale 5: 8073–8083. 23877169 10.1039/c3nr01429k

[36] Garcia M (2011) Surface plasmons in metallic nanoparticles: fundamentals and applications. J Phys D Appl Phys 44: 283001.

[37] Liu Z, Hou W, Pavaskar P, Aykol M, Cronin SB (2011) Plasmon resonant enhancement of photocatalytic water splitting under visible illumination. Nano Lett 11: 1111–1116. 21319840 10.1021/nl104005n

[38] Wang P, Huang B, Dai Y, Whangbo MH (2012) Plasmonic photocatalysts: harvesting visible light with noble metal nanoparticles. Phys Chem Chem Phys 14: 9813–9825. 22710311 10.1039/c2cp40823f

[39] Yu JG, Yu HG, Cheng B, Zhao XJ, Yu JC, et al (2003) The effect of calcination temperature on the surface microstructure and photocatalytic activity of TiO_2_ thin films prepared by liquid phase deposition. J Phys Chem B 107: 13871–13879.

[40] Cong Y, Zhang J, Chen F, Anpo M (2007) Synthesis and characterization of nitrogen-doped TiO_2_ nanophotocatalyst with high visible light activity. J Phys Chem C 111: 6976–6982.

[41] Hoang S, Guo S, Hahn NT, Bard AJ, Mullins CB (2011) Visible light driven photoelectrochemical water oxidation on nitrogen-modified TiO_2_ nanowires. Nano Lett 12: 26–32. 22112010 10.1021/nl2028188

[42] Tian B, Li C, Gu F, Jiang H (2009) Synergetic effects of nitrogen doping and Au loading on enhancing the visible-light photocatalytic activity of nano-TiO_2_ . Catal Commun 10: 925–929.

[43] Zhang J, Wang Y, Zhang J, Lin Z, Huang F, et al (2013) Enhanced photocatalytic hydrogen production activities of Au-loaded ZnS flowers. ACS Appl Mater Inter 5: 1031–1037. 10.1021/am302726y 23320503

[44] Zhang P, Shao C, Li X, Zhang M, Zhang X, et al (2012) In situ assembly of well-dispersed Au nanoparticles on TiO_2_/ZnO nanofibers: a three-way synergistic heterostructure with enhanced photocatalytic activity. J Hazard Mater 237: 331–338. 22975259 10.1016/j.jhazmat.2012.08.054

[45] Min Y, He G, Li R, Zhao W, Chen Y, et al (2013) Doping nitrogen anion enhanced photocatalytic activity on TiO_2_ hybridized with graphene composite under solar light. Sep Purif Technol 106: 97–104.

[46] Wang H, Zhang C, Liu Z, Wang L, Han P, et al (2011) Nitrogen-doped graphene nanosheets with excellent lithium storage properties. J Mater Chem 21: 5430–5434.

[47] Bessekhouad Y, Brahimi R, Hamdini F, Trari M (2012) Cu_2_S/TiO_2_ heterojunction applied to visible light Orange II degradation. J Photoch Photobio A 248: 15–23.

[48] Xin B, Ren Z, Hu H, Zhang X, Dong C, et al (2005) Photocatalytic activity and interfacial carrier transfer of Ag-TiO_2_ nanoparticle films. Appl Surf Sci 252: 2050–2055.

