# Supplementary material for: Enhanced Photocatalytic Activity for H2 Evolution under Irradiation of UV–Vis Light by Au-Modified Nitrogen-Doped TiO2
Source: PLoS One. 2014 Aug 4;9(8):e103671. doi: 10.1371/journal.pone.0103671 (PMC4121273; doi:10.1371/journal.pone.0103671)
Supplement: File S1 — (DOCX) [file pone.0103671.s001.docx]

Figure S1. XRD patterns of P25, TNT, TiO_2_, N-TiO_2_ and Au/N-TiO_2_. Asterisk, rhombus, and open star denote rutile, anatase, and Au, respectively.

**Au (111)**

**d = 0.239 nm**

**TiO_2_ (101)**

**d = 0.360 nm**

(b)

(a)

**Au**


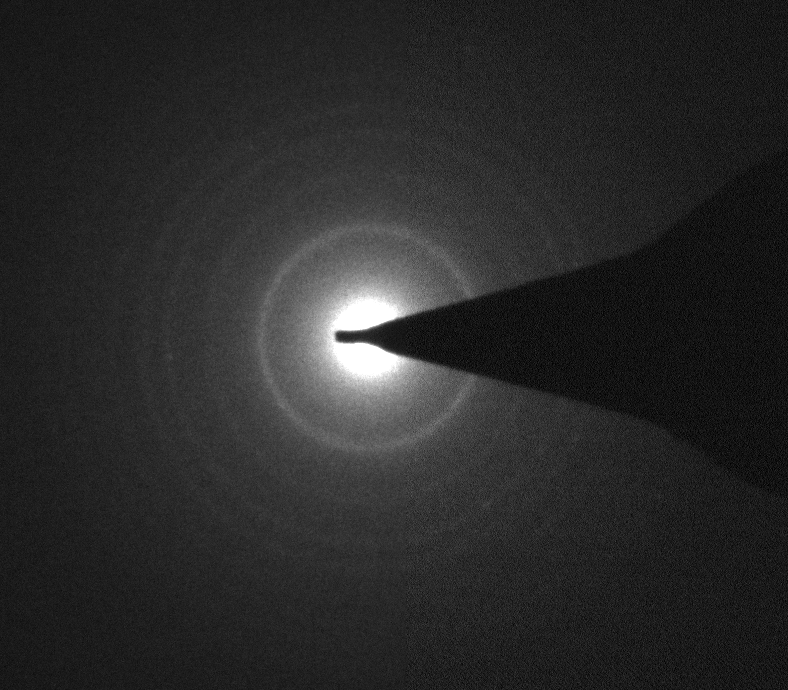


**(101)**

**(004)**

**(200)**

Figure S2. (a) TEM image of as synthesized Au/N-TiO_2_, inset in the lower right corner is the SAED pattern of anatase TiO_2_ and (b) HRTEM image and (c) EDX spectrum of Au/N-TiO_2_.

Figure S3. XPS spectra of (a) Au/N-TiO_2_ and core level spectra of (b) O 1s, (c) N 1s, and (d) Au 4f.

Figure S4. UV–vis diffuse reflectance spectra of TiO_2_, Au/TiO_2_, N-TiO_2_, and Au/N-TiO_2_.

Figure S5. PL emission spectra of TiO_2_, Au/TiO_2_, N-TiO_2_, and Au/N-TiO_2_ under the irradiation of 254 nm.

Figure S6. Photocatalytic activity for water splitting under the irradiation of (a) UV and (b) UV–vis light.

Figure S7. The repeated hydrogen evolution tests over Au/N-TiO_2_ under UV-vis light in (a) pure water and (b) methanol/water solution with purging of Ar in every 8 h.

Figure S8. Photocurrents of TiO_2_, N-TiO_2_, Au/TiO_2_, and Au/N-TiO_2_ electrodes at zero bias voltage irradiated with (a) UV (*λ* = 254 nm) and (b) visible light (λ > 400 nm) for 20 s.

Figure S9. EIS Nyquist plots for Au/N-TiO_2_ in dark and under the irradiation of visible light. Inset is the suggested equivalent circuit and the fitting results for Au/N-TiO_2_. Rs and Rct are the electrolyte and electron-transfer resistance, respectively. CPE is the constant phase element, which also represents the double layer capacitance. Ws is the Warburg impedance. Y_0_ is the value of admittance and expresses a reciprocal relationship to the Warburg coefficient, which is able to predict the Warburg impedance and diffusion coefficient.





Figure S10. Schematic illustration of Au/N-TiO_2_ for water splitting under the irradiation of (a) UV and (b) visible light. Pathway I denotes the generation of charge carriers in TiO_2_. Pathway II represents the reversible electron transfer between charged diamagnetic N_b_^-^ and neutral paramagnetic N_b_^•^, and the excitation of electrons into the conduction band. Pathway III shows the acceleration of photo-induced electrons transfer by Au loading. Pathway IV denotes the SPR effect of loaded Au nanoparticles.

**Supporting Information Legends**

Figure S1. XRD patterns of P25, TNT, TiO_2_, N-TiO_2_ and Au/N-TiO_2_. Asterisk, rhombus, and open star denote rutile, anatase, and Au, respectively.

Figure S2. (a) TEM image of as synthesized Au/N-TiO_2_, inset in the lower right corner is the SAED pattern of anatase TiO_2_ and (b) HRTEM image and (c) EDX spectrum of Au/N-TiO_2_.

Figure S3. XPS spectra of (a) Au/N-TiO_2_ and core level spectra of (b) O 1s, (c) N 1s, and (d) Au 4f.

Figure S4. UV–vis diffuse reflectance spectra of TiO_2_, Au/TiO_2_, N-TiO_2_, and Au/N-TiO_2_.

Figure S5. PL emission spectra of TiO_2_, Au/TiO_2_, N-TiO_2_, and Au/N-TiO_2_ under the irradiation of 254 nm.

Figure S6. Photocatalytic activity for water splitting under the irradiation of (a) UV and (b) UV–vis light.

Figure S7. The repeated hydrogen evolution tests over Au/N-TiO_2_ under UV-vis light in (a) pure water and (b) methanol/water solution with purging of Ar in every 8 h.

Figure S8. Photocurrents of TiO_2_, N-TiO_2_, Au/TiO_2_, and Au/N-TiO_2_ electrodes at zero bias voltage irradiated with (a) UV (*λ* = 254 nm) and (b) visible light (λ > 400 nm) for 20 s.

Figure S9. EIS Nyquist plots for Au/N-TiO_2_ in dark and under the irradiation of visible light. Inset is the suggested equivalent circuit and the fitting results for Au/N-TiO_2_. Rs and Rct are the electrolyte and electron-transfer resistance, respectively. CPE is the constant phase element, which also represents the double layer capacitance. Ws is the Warburg impedance. Y_0_ is the value of admittance and expresses a reciprocal relationship to the Warburg coefficient, which is able to predict the Warburg impedance and diffusion coefficient.

Figure S10. Schematic illustration of Au/N-TiO_2_ for water splitting under the irradiation of (a) UV and (b) visible light. Pathway I denotes the generation of charge carriers in TiO_2_. Pathway II represents the reversible electron transfer between charged diamagnetic N_b_^-^ and neutral paramagnetic N_b_^•^, and the excitation of electrons into the conduction band. Pathway III shows the acceleration of photo-induced electrons transfer by Au loading. Pathway IV denotes the SPR effect of loaded Au nanoparticles.
